# The Relationship between Vitamin D Status and Allergic Diseases in New Zealand Preschool Children

**DOI:** 10.3390/nu8060326

**Published:** 2016-06-01

**Authors:** Carolyn Cairncross, Cameron Grant, Welma Stonehouse, Cath Conlon, Barry McDonald, Lisa Houghton, Darryl Eyles, Carlos A. Camargo, Jane Coad, Pamela von Hurst

**Affiliations:** 1 School of Food and Nutrition, Massey University, Auckland 0632, New Zealand; C.t.cairncross@massey.ac.nz (C.Ca.); c.conlon@massey.ac.nz (C.Co.); j.coad@massey.ac.nz (J.C.); 2 Department of Paediatrics, University of Auckland and General Paediatrics, Auckland 1010, New Zealand; cc.grant@auckland.ac.nz; 3 Starship Children’s Hospital, Aucklan 1023, New Zealand; 4 CSIRO Food and Nutrition, Adelaide 5000, Australia; welma.stonehouse@csiro.au; 5 Institute of Natural and Mathematical Sciences, Massey University, Auckland 0632, New Zealand; b.mcdonald@massey.ac.nz; 6 Department of Human Nutrition, University of Otago, Dunedin 9016, New Zealand; lisa.houghton@otago.ac.nz; 7 Queensland Brain Institute, University of Queensland, Brisbane 4072, Australia; d.eyles@uq.edu.au; 8 Queensland Centre for Mental Health Research, Brisbane 4072, Australia; 9 Department of Emergency Medicine, Massachusetts General Hospital, Harvard Medical School, Boston, MA 02114, USA; ccamargo@partners.org

**Keywords:** vitamin D, children, allergic disease, New Zealand

## Abstract

Recent research on vitamin D in young children has expanded from bone development to exploring immunomodulatory effects. Our aim was to investigate the relationship of vitamin D status and allergic diseases in preschool-aged children in New Zealand. Dried capillary blood spots were collected from 1329 children during late-winter to early-spring for 25(OH)D measurement by LC-MS/MS. Caregivers completed a questionnaire about their child’s recent medical history. Analysis was by multivariable logistic regression. Mean 25(OH)D concentration was 52(SD19) nmol/L, with 7% of children <25 nmol/L and 49% <50 nmol/L. Children with 25(OH)D concentrations ≥75 nmol/L (*n* = 29) had a two-fold increased risk for parent-report of doctor-diagnosed food allergy compared to children with 25(OH)D 50–74.9 nmol/L (OR = 2.21, 1.33–3.68, *p* = 0.002). No associations were present between 25(OH)D concentration and presence of parent-reported eczema, allergic rhinoconjunctivitis or atopic asthma. Vitamin D deficiency was not associated with several allergic diseases in these New Zealand preschool children. In contrast, high 25(OH)D concentrations were associated with a two-fold increased risk of parental-report food allergy. This increase supports further research into the association between vitamin D status and allergic disease in preschool children.

## 1. Introduction

Vitamin D research in young children has previously concentrated on skeletal health including rickets. Recent discoveries of an immune modulation role for vitamin D has led to the investigation of associations of vitamin D status with a number of immune mediated childhood illnesses, including auto-immune, atopic and infectious conditions [1,2,3]. Few cross-sectional studies have explored the relationship of allergic diseases with vitamin D status, particularly in preschool aged children.

Morbidity from childhood allergic diseases can be high and includes diminished quality of life and increased health costs for the child, their family and the community [4]. New Zealand children have amongst the highest rates of eczema, allergic rhinoconjunctivitis and asthma in the world [5,6]. Exaggerated immune responses to certain food proteins and associated anaphylaxis have increased over the past two decades in neighbouring Australia and worldwide [7].

The immune-modulatory actions of vitamin D affect both the innate and adaptive components of the immune system, and may offer potential targets for prevention or treatment of allergic diseases [8]. Research suggests the active form of vitamin D, 1,25(OH)_2_D, contributes to the immune system through mechanisms that modulate immunologic tolerance, suppress pro-allergic immune responses, maintain the integrity of the epithelial barrier and reduce susceptibility to infection [9,10,11]. Animal model studies show evidence for a vitamin D mediated enhanced T helper 2 (Th_2_) cell antigen response by two pathways; firstly by shifting the T lymphocyte balance toward Th_2_ cells through the inhibition of Th_1_ cells and secondly, through increased production of Th_2_ cells through the differentiation of naive T-Cells into Th_2_ cells [12,13]. A down regulation of the Th_2_-driven immune response inhibits skin barrier function [14] and the production of the Th_17_ cytokines allied with asthma severity [15]. For food allergy, it has been hypothesised that the combined effect of vitamin D deficiency and infections decreases the intestinal barrier integrity [16], which allows low doses of food protein the ability to infiltrate the immune system, leading to increased immunoglobulin E (IgE) production by beta-cells and allergenic Th_2_ immune responses [17].

With New Zealand preschool children having a high burden of allergic disease and living in a country with abundant sunshine yet high population rates of vitamin D deficiency, the aim of this study was to investigate the relationship between vitamin D status and prevalence of allergic diseases—eczema, food allergy, allergic rhinoconjunctivitis and atopic asthma—in a self-selected sample of New Zealand preschool children aged 2 to <5 years using 25(OH)D concentration measured on dried blood spot.

## 2. Materials and Methods

### 2.1. Study Design and Participants

Participants (1329) in this cross-sectional study were preschool children aged between 2 and <5 years of age recruited from 17 cities and towns throughout New Zealand (latitude 35°–46°S). Ethical approval was granted by the Health and Disability Ethics Committee, Northern Region, reference number NTX/12/04/036, and a parent or caregiver of each participant provided written informed consent for the participation of their child in the study.

### 2.2. Data Collection

Blood samples were obtained from enrolled children by trained staff at 49 participating community pharmacies as well as the nutrition clinics at Massey University, Auckland, and the University of Otago, Dunedin from August to October (late-winter to early-spring), 2012. A capillary blood sample from a finger prick was obtained from each child using paediatric lancets. Blood was dropped onto a pre-stamped circle on Whatman 305 filter paper and allowed to air dry for a minimum of one hour. All samples were stored at room temperature prior to analysis at Queensland Brain Institute, Brisbane, Australia, for measurement of 25(OH)D concentration.

Parents or caregivers completed a 59-item questionnaire, either online (www.surveymonkey.com) or paper format, on basic demographics, recent health history and factors associated with vitamin D status. All identified ethnicities for the child were listed by caregivers, with ethnicity then assigned using the following prioritisation; Māori, then Pacific, then Other, then European [18]. Caregivers rated their child’s skin colour on a six-point scale (very fair, fair, medium, olive, dark and very dark). This was then collapsed into 3 skin colour categories (very fair-fair, medium, olive dark-very dark).

Caregivers were asked if their child attended formal preschool education facilities such as daycare, preschool or kindergarten. For dietary sources of vitamin D, use of supplements containing vitamin D and cod liver oil was determined, along with consumption of milks. A list of common milks and those low-fat milks fortified with vitamin D was provided, along with a free-text option for other milks consumed. Vitamin D fortified cow’s milk formula marketed to young children was defined as toddler milk. Maternal education was classified as no secondary qualifications, secondary qualifications or post-secondary qualifications.

### 2.3. Measurement of 25(OH)D Concentrations

The analytical method, in brief, involves a derivatising agent combined with liquid chromatography tandem mass spectrometry [19]. The assay measured both 25(OH)D_2_ and 25(OH)D_3_, with results described as total 25(OH)D. Precise 3.2 mm whole blood punches, equivalent to 3.2 µL of whole blood, were obtained from each dried blood spot (DBS). As 25(OH)D is excluded from erythrocytes, the following equation was used to correct for the haematocrit fraction in serum of children in the preschool age group (0.41 for girls and 0.45 for boys) [20]:

Plasma [25(OH)D] (nM) = DBS [25(OH)D] (nM) /(1 − haematocrit fraction)(1)

### 2.4. Disease Outcome Measures

Eczema, allergic rhinoconjunctivitis and atopic asthma were defined by parental report using the modules of the ISAAC questionnaire for children aged 6–7 years [5]. Eczema was defined as an itchy rash in the last 12 months affecting various body sites. Allergic rhinoconjunctivitis was defined as sneezing or runny nose symptoms in the last 12 months accompanied by itchy watery eyes. Atopic asthma was defined as wheezing or whistling in the chest in the last 12 months combined with report of eczema and/or food allergy. Doctor diagnosed food allergy was defined as a caregiver answering positively to the initial question “has your child ever had food allergy?” and then subsequently to the question “was your child’s allergy confirmed or diagnosed by a doctor?”

### 2.5. Statistical Analyses

Dried blood spot 25(OH)D concentrations were categorised into 4 groups; <25, 25 to 49.9, 50 to 74.9 and ≥75 nmol/L. This grouping is consistent with the variation in definitions for vitamin D deficiency [21]. The association between the presence of each disease outcome and the categorised 25(OH)D concentration was investigated using univariable and multivariable logistic regression analysis, and reported using OR and 95%CI. The reference 25(OH)D category used in logistic regression was 50 to 74.9 nmol/L [21]. Analysis by linear regression was also carried out. As the results did not differ, only the logistic regression results are presented. Differences between disease outcome in mean 25(OH)D concentration were determined using Independent *t*-tests.

Demographic and environmental risk factors were considered as potential confounders in a logistic regression model for each disease outcome. Those confounding variables with a *p* value of < 0.20 in one predictor model analysis were entered into the logistic regression model. Those variables of non-significance were removed singly using a backwards stepwise method. The resultant confounders varied according to disease outcome. If a variable was a significant confounder for one disease outcome it was included in the multivariable logistic regression analysis for all disease outcomes to avoid unintentional bias in estimation of the relationship between disease outcome and dried blood spot 25(OH)D concentration. Each disease outcome model was also subsequently adjusted for confounding variables specific to each condition. Independent associations of 25(OH)D concentration with diseases outcomes were described using odds ratios (OR) and 95% confidence intervals (CI).

Interaction effects were tested for each disease outcome. No significant interactions were seen. The presence of interaction was assessed using two methods, firstly through logistic regression models where the variable was multiplied by 25(OH)D and significance calculated, and secondly, through generation of scatterplot of response* versus* 25(OH)D with smoother lines.

All analyses were performed with the Statistical Package for the Social Sciences (SPSS 21.0 (IBM Corp., Armonk, NY, USA)). All tests with statistical significance were determined by *p* < 0.05.

## 3. Results

### 3.1. Participant Characteristics

The participant characteristics are presented in Table 1. The mean age of the children was 41 months (range 24 to 60 months), with the majority being of NZ European ethnicity (70%), attending daycare (84%) and having a mother with higher educational qualifications (81%). Almost one-third of children (29%) consumed a supplement containing vitamin D or cod liver oil while 6% reported drinking toddler milk. The mean dried blood spot 25(OH)D concentration was 52 nmol/L (range 5 to 173 nmol/L).

Stepwise regression modelling confirmed 7 factors to be associated with disease outcome. These 7 factors were included as potential confounders in all disease outcome analyses. These factors were gender, ethnicity, skin colour, daycare attendance, usage of vitamin D supplements, consumption of toddler milk (vitamin D fortified cow’s milk formula marketed to young children) and maternal education.

### 3.2. Disease Outcomes

There was a variation in prevalence of the allergic diseases in the participants of this study compared those from national samples of children aged 6 to 7 years. Prevalence was higher for eczema (23% *vs*. 17%) and atopic asthma (32% *vs*. 22%), while similar for allergic rhinoconjunctivitis (10% *vs*. 11%) [5]. Rates of food allergy in New Zealand have yet to be determined, however the 12% prevalence of parental-report doctor-diagnosed food allergy in this study reflects epidemiological studies from neighbouring Australia which estimate food allergy rates of approximately 10% [22,23] (Table 2).

No significant difference in mean 25(OH)D concentration was seen for children with and without eczema (53 *vs*. 52 nmol/L, *p* = 0.50), allergic rhinoconjunctivitis (52 *vs*. 52 nmol/L, *p* = 0.94) and atopic asthma (52 *vs*. 52 nmol/L, *p* = 0.98). In contrast, children with food allergy had a higher mean 25(OH)D concentration than those who did not (56 *vs*. 52 nmol/L, *p* = 0.007).

In comparison with children with a 25(OH)D concentration of 50 to 74.9 nmol/L, the odds of doctor diagnosed food allergy were increased for children with a 25(OH)D concentration ≥75 nmol/L (OR 2.21, 95%CI 1.33–3.68, *p* = 0.002). In comparison with children with a 25(OH)D concentration of 50 to 74.9 nmol/L, the odds of eczema, allergic rhinoconjunctivitis or atopic asthma were neither increased nor decreased with children with higher or lower 25(OH)D concentrations (Table 2).

## 4. Discussion

In this cross-sectional study of 1329 children aged 2 to <5 years, higher concentrations of 25(OH)D were associated with an increased risk of parent-reported doctor-diagnosed food allergy. No association was evident between 25(OH)D concentration and the odds of eczema, allergic rhinoconjunctivitis and atopic asthma.

Other observational studies that have enrolled preschool aged children and investigated associations of vitamin D status with eczema and asthma report contradictory findings. Increased eczema prevalence and severity have been associated with both lower [24,25,26,27,28] and higher [29] 25(OH)D concentrations. An inverse correlation of 25(OH)D concentration and asthma prevalence in children attending hospital in Denver [30] and Sweden [31] is in contrast to the lack of association of 25(OH)D and asthma found in Massachusetts [32], while mean 25(OH)D concentrations were lower in children with asthma compared to controls in Egyptian [33], Quatari [34] and Turkish hospitals [35]. In recent supplementation trials of vitamin D and asthma parameters, no change was seen in Irish school children compared to placebo [36] while improvements were noted in Japanese schoolchildren [37]. Direct comparison of the data from our study that shows no association of 25(OH)D with the odds of eczema and atopic asthma is difficult because the inclusion of infants and adolescents in these other study samples, differences in diagnostic criteria used to define eczema and asthma and in the prevalence of these diseases in the populations studied.

The diagnosis of asthma in very young children can be challenging and lack of diagnosis by a doctor is a limitation of the current study. As a proxy of doctor diagnosis, parents were asked “which asthma medicines has your child used?” as these medications are required to be prescribed by a medical professional. Results of logistic regression analysis for the children with atopic asthma who reported asthma medication usage also found no association with 25(OH)D concentration. Diagnosis of asthma by a medical professional is recommended for future research studies involving preschool children.

To date few studies have directly investigated associations between disease prevalence and 25(OH)D concentration. In infants, vitamin D insufficiency is associated with an increased risk of challenge proven peanut/egg allergy [38] and vitamin D deficiency increased the risk of sensitization to food allergens, particularly to milk and wheat [39]. These findings are in contrast to our study finding of increased risk of food allergy and 25(OH)D concentrations ≥75 nmol/L. This finding needs to be considered in light of the imprecise nature of parental-report of this cross-sectional study compared to clinical measurement criteria, the differences in ages, ethnicity and country of origin of the enrolled children and dried blood spot measurement of 25(OH)D compared to serum 25(OH)D. In addition, genotype was not assessed in our study, with recent evidence polymorphisms associated with vitamin D binding protein concentrations reduce the relationship between low 25(OH)D concentrations and food allergy [40].

Conflicting findings have been reported from observational studies of the relationship between vitamin D status and allergic rhinoconjunctivitis. There was no association between 25(OH)D concentration and allergic rhinitis in studies of adults in Korea [41] and children in Qatar [42] while higher rates of vitamin D deficiency were observed in Iranian adults with allergic rhinitis compared to population controls [43]. In contrast, for children and adults in the US, the prevalence of allergic rhinitis increases as serum 25(OH)D concentration increased [44]. Birth cohort studies report both inverse and null associations of early-life measures of vitamin D status with childhood allergic rhinitis [45,46,47]. The results of this study are similar to those in Korea [41] and Qatar [42]. Although non-significant (*p* = 0.05), the observation of a small, increased risk of allergic rhinoconjunctivitis for children with 25(OH)H concentrations below 25 nmol/L compared to the reference category reflect findings from Iranian adults [43] and birth cohort studies [46].

This was a relatively large study of free-living healthy children enrolled from a number of centres throughout the country. Study limitations include the cross-sectional nature, which can identify associations but not establish causality and the proportion of children with 25(OH)D < 25 nmol/L. There is no capacity within a study of this design to confirm the presence or absence or severity of each of these parental-reported diagnoses. Use of the ISAAC questionnaire for children aged 6–7 years of age in this preschool age group may affect the prevalence of disease, for example, the challenging diagnosis of asthma in very young children compared to older children. The diseases were not confirmed by the diagnosis of a doctor nor the presence of intermittent or perennial symptoms. Thus, the estimation of prevalence of each allergic disease will be imprecise due to the nature of parental recall and report. This imprecise measurement is likely to reduce the capacity to identify any associations between vitamin D status and disease risk. Our study finding of increased risk of food allergy for children with 25(OH)D ≥ 75 nmol/L needs to be considered in light of this study limitation and the small number of children with doctor-diagnosed food allergy, as it is not known whether high 25(OH)D concentrations cause a higher prevalence of food allergy or whether presence of food allergy and higher 25(OH)D concentration are due to a separate unrelated factor. Although the use of dried blood spots to measure 25(OH)D concentration enabled a large sample size in a vulnerable population, there may be limitations in comparison to studies using serum blood samples. However, good correlation and reliability have been reported between 25(OH)D concentrations from dried blood spot and serum samples in previous studies [48,49,50]. Bias in our recruitment strategy of self-selected participants through email resulted in a sample group of higher socioeconomic status and maternal education level than has been reported nationally [51,52]. The higher numbers of enrolled children with eczema and asthma compared to national samples may have been due bias in our advertising material which sought children to investigate the links between vitamin D and these diseases, and limits generalisation of our findings to the overall preschool population.

Findings from birth cohort studies and from animal models suggest that the immune system tolerance and microbial bacterial environment may be sensitive to vitamin D status at a critical time period in pregnancy and/or early life, both to deficiency and excess [16,53]. Longitudinal study designs are necessary to fully understand the relationship between vitamin D status and allergic diseases, ideally from pregnancy onwards. Randomised controlled trials are needed to determine if the risk of atopic disease is altered by vitamin D supplementation, such as the current VITALITY trial in young children [54].

## 5. Conclusions

In this study of 1329 children aged between 2 and <5 years, there was no association between vitamin D status and parental-reported prevalence of eczema, atopic asthma or allergic rhinoconjunctivitis. Children with 25(OH)D concentrations ≥75 nmol/L had a two-fold increased risk of parental-reported, doctor-diagnosed food allergy. With the high rates of allergic diseases in young New Zealand children, further investigation into the relationship of these diseases with vitamin D status should be a research priority.

## Figures and Tables

**Table 1:** Participant characteristics by dried blood spot 25(OH)D categories.

Variable	*n* (%)	25(OH)D Categories (nmol/L) *n* (%)			
		<25	25 to 49.9	50 to 74.9	≥75
Total	1329	86 (7)	556 (42)	541 (41)	146 (11)
Mean (95%CI) (nmol/L)	52 (19)	19 (18–20)	39 (39–40)	61 (60–61)	87 (85–90)
Median	51	20	40	60	82
Interquartile Range (IQ)	25	7	12	12	14
Age (years)					
2 years old	496 (37)	30 (6)	223 (45)	183 (37)	60 (12)
3 years old	448 (34)	25 (6)	180 (40)	194 (43)	49 (11)
4 years old	385 (29)	31 (8)	153 (40)	164 (43)	37 (10)
Gender					
Female	648 (49)	51 (8)	296 (46)	248 (38)	53 (8)
Male	681 (51)	35 (5)	260 (38)	293 (43)	93 (14)
Ethnicity					
NZ European	926 (70)	32 (3)	378 (41)	401 (43)	115 (12)
Māori	174 (13)	16 (9)	74 (43)	66 (38)	18 (10)
Pacific	44 (3)	10 (23)	21 (48)	12 (27)	1 (2)
Other non-European	185 (14)	28 (15)	83 (45)	62 (34)	12 (7)
Skin colour—parental report					
Very fair—fair	767 (58)	27 (4)	339 (44)	313 (41)	88 (11)
Medium	445 (33)	29 (7)	172 (39)	193 (43)	51 (11)
Olive—very dark	17 (9)	30 (26)	45 (38)	35 (30)	7 (6)
Attend formal daycare					
Yes	1137 (86)	71 (6)	464 (41)	473 (42)	129 (11)
No	192 (14)	15 (8)	92 (48)	68 (35)	17 (9)
Vitamin D supplement use *					
Yes	253 (19)	6 (2)	78 (31)	131 (52)	38 (15)
No	1076 (81)	80 (7)	478 (44)	410 (38)	108 (10)
Drinks toddler milk					
Yes	80 (6)	0 (0)	20 (25)	44 (55)	16 (20)
No	1249 (94)	86 (7)	536 (43)	497 (40)	130 (10)
Maternal-education qualifications					
No secondary	59 (4)	20 (34)	23 (39)	13 (22)	3 (5)
Secondary	98 (15)	10 (5)	93 (47)	77 (39)	18 (9)
Post-secondary	1072 (81)	56 (5)	440 (41)	451 (42)	125 (12)

*: Includes cod liver oil.

**Table 2:** Prevalence and odds ratios for allergic diseases by dried blood spot 25(OH)D concentration.

Allergic Disease 25(OH)D Concentration (nmol/L)	*n*	Unadjusted		Adjusted *	
		OR (95%CI)	*p* Value	OR (95%CI)	*p* Value
Atopic asthma (*n* = 420)					
<25	25	0.96 (0.58, 1.58)	0.87	0.81 (0.46, 1.41)	0.45
25 to 49.9	184	1.16 (0.90, 1.49)	0.26	1.19 (0.91, 1.55)	0.21
50 to 74.9	162	1.00	-	1.00	-
≥75	49	1.18 (0.80, 1.75)	0.40	1.13 (0.76, 1.69)	0.55
Eczema (*n* = 299)					
<25	15	0.78 (0.43, 1.42)	0.42	0.66 (0.34, 1.29)	0.22
25 to 49.9	132	1.15 (0.87, 1.53)	0.33	1.20 (0.92, 1.55)	0.26
50 to 74.9	115	1.00	-	1.00	-
≥75	37	1.26 (0.82, 1.93)	0.29	1.23 (0.79, 1.89)	0.36
Food allergy(*n* = 153)					
<25	4	0.45 (0.16, 1.27)	0.13	0.45 (0.15, 1.36)	0.16
25 to 49.9	67	1.26 (0.86, 1.85)	0.23	1.39 (0.93, 2.06)	0.21
50 to 74.9	53	1.00	-	1.00	-
≥75	29	2.82 (1.39, 3.75)	<0.001	2.21 (1.33, 3.68)	0.002
Allergic rhinoconjunctivitis (*n* = 139)					
<25	14	1.91 (1.01, 3.63)	0.05	1.82 (0.89, 3.72)	0.10
25 to 49.9	56	1.10 (0.74, 1.64)	0.64	1.14 (0.75, 1.72)	0.54
50 to 74.9	50	1.00	-	1.00	-
≥75	19	1.47, (0.84, 2.58)	0.18	1.45 (0.82, 2.58)	0.20

*: Adjusted for gender, ethnicity, skin colour, daycare attendance, vitamin D supplement and toddler milk usage, maternal education level. OR odds ratio, CI confidence interval.

## Abbreviations

The following abbreviations are used in this manuscript:

LC-MS/MS	liquid chromatography with tandem mass-spectrometer
25(OH)D	25 hydroxyvitamin D
1-25(OH)_2_D	1,25 di-hydroxyvitamin D
Th_2_	T helper 2 cells
IgE	immunoglobulin E
DBS	dried blood spot
